# Nursing the Baby

**Published:** 1920-11-27

**Authors:** 


### Nursing the Baby.

A LITTLE storm of protest has been aroused by the statements of a writer in one of the London evening newspapers as to the refusal of the modern mother to nurse her baby. "I have always taken an interest in this subject," declares Mrs. Henderson, wife of Major V. L. Henderson, M.P., "and have had many opportunities of discussing it with maternity nurses and others who occupy themselves with the care of babies, and I have only heard of very isolated cases where mothers will not nurse their babies, though capable of doing so. I grant that the present nerve-racking life we lead has rendered many more mothers incapable of nursing their babies than formerly, but I cannot agree with the suggestion that a mother shirks her duty because of the pain. Considering what she has suffered, it is hardly fair to accuse her of being afraid of pain. . . . I think many mothers will agree with me that one of the happiest times of a woman's life is when she is nursing her baby."

That is a natural complaint which one often hears, but is it not a fact that the habit of rearing babies with the bottle instead of at the breast is distinctly increasing among certain classes of society—not because the mothers are incapable of producing the milk, but deliberately, for reasons not altogether unselfish? We should like to know the opinion of experienced general practitioners on this point. The argument advanced in the article in the *Evening Standard* to which exception has been taken was in the form of a condemnation of feminine education on account of its relationship to the decline in breast-rearing mothers. It was not, perhaps, entirely safe ground to take.

